# Goal‐oriented practices in youth mental health and wellbeing settings: A scoping review and thematic analysis of empirical evidence

**DOI:** 10.1111/papt.12564

**Published:** 2024-12-13

**Authors:** Jenna Jacob, Lori Wozney, Hanne Weie Oddli, Charlie Duncan, Jill Chorney, Debbie Emberly, Duncan Law, Sharon Clark, Sofie Heien, Leah Boulos, Mick Cooper

**Affiliations:** ^1^ Anna Freud London UK; ^2^ University College London London UK; ^3^ IWK Health Halifax Nova Scotia Canada; ^4^ Department of Psychology University of Oslo Oslo Norway; ^5^ British Association for Counselling and Psychotherapy Lutterworth UK; ^6^ School of Psychology University of Roehampton London UK; ^7^ Department of Psychiatry Dalhousie University London Canada; ^8^ MindMonkey Associates Halifax Nova Scotia UK

**Keywords:** child and adolescent mental health, goal‐oriented practice, scoping review

## Abstract

**Introduction:**

Goal‐oriented practices involve practitioners working collaboratively with clients to identify, develop and focus on objectives for the therapeutic work. It has been suggested that the key mechanism underpinning goal‐oriented practices with young people is the development of epistemic trust via the foundation of open communication, along with shared decision‐making: including young people in decisions about their care. However, goal‐oriented work in practice is variable in scope and content, with no research consensus on what it entails, the mechanisms of change and reported outcomes.

**Method:**

This research aims to map the extent, range and nature of the evidence‐base for goal‐oriented practices, including gaps, through a synthesis of the available empirical evidence from the past 20 years. A scoping review of 9783 studies published from 2003 onwards was conducted.

**Results:**

In total, 116 studies were identified for inclusion in the review, focusing on goal‐oriented practices specifically related to the mental health and wellbeing of children and young people aged 0–18 (including caregivers as relevant). Alongside presentation of the key elements of the included studies, three themes were developed relating to the features of mental health and wellbeing goal‐oriented practice in the contexts of the studies: Conceptual and Empirical Constructs of Goal‐Oriented Practices, Quality and Making ‘Good’ Goals, and The Socio‐Cultural Contexts of Goal‐Oriented Practice.

**Conclusion:**

Several areas for future research are identified that will build on this evidence, and further understanding in this area. Work towards the development of best practice principles will move practice towards transparency in the understanding and delivery of goal‐oriented practices.

Goal‐oriented practice is defined as ‘any therapeutic encounter that works towards helping the person move towards what they want to get from therapy’ (Cooper & Law, 2018, p. 161). Goal‐oriented practices encapsulate a range of therapeutic tasks that can be used with adult and child clients and include goal‐setting (‘the process of identifying and establishing goals’), goal‐tracking (‘the evaluation of clients’ progress towards their goals') and goal discussion (‘any process in which client and therapist collaboratively talk about the goals for therapy’; Cooper & Law, 2018, p. 3). In goal‐oriented practices, practitioners work collaboratively with clients to identify, develop and focus on objectives for the therapeutic work. For instance, the client and therapist may agree that the work should focus on helping the client overcome feelings of loss or readying the client for a new relationship (Cooper & Law, 2018). Goal‐oriented practices involve a continuous feedback loop of striving towards the desired endpoint, which may also act as a self‐regulation strategy for clients (Harkin et al., 2016). Goals themselves have been described as representing desired endpoints that fill a perceived gap between the current and desired status (Austin & Vancouver, 1996). In goal‐tracking, goals are typically recorded and monitored through an individualized outcome measure, such as the Goal‐Based Outcomes tool (GBO; Law, 2013, 2019) and the Goals Form (Cooper & Xu, 2023), although others have been used in therapeutic settings (for an overview see Lloyd et al., 2019).

Given the heterogeneity and developmental complexity of youth‐aged clients, including age‐related differences regarding understanding the concept of goals and the use of goal setting in everyday life, there is a need to examine goal‐oriented practices in mental health contexts with youth as a distinct group, separate from adults. Although goal‐oriented practices are a common part of many psychological therapies for youth (Weisz et al., 2011), how and why specific goal‐oriented practices may have a positive impact on mental health and wellbeing, and which therapeutic mechanisms account for these changes, are not well‐established for this population (Cairns et al., 2015). Further, goal collaboration and agreement has been described as missing from a great deal of work with youth‐aged clients, even though youth have described goal‐oriented practices as a ‘social contract’ forming the basis of their therapeutic work (Hartley et al., 2022). Goal‐oriented practice as a therapeutic technique may represent a common element across therapies, however, there is no clearly defined and agreed upon definition of goal‐oriented practices that spans different modalities of support. In addition, specific elements of goal‐related practices may mediate or moderate the relative efficacies of different therapies.

Emerging research around these mechanisms of change suggests that goal‐oriented practices may help build the therapeutic alliance with youth (Jacob et al., 2022) primarily through creating a shared understanding, a shared language, an agreed way of working, and focus on centring youth's important areas of change, providing a sense of autonomy. It has also been suggested that the key mechanism underpinning goal‐oriented practices with youth is the development of ‘epistemic trust’ via the open communication the approach engenders, along with shared decision‐making: including youth in decisions about their care (Law, 2022). Epistemic trust ‘describes the willingness to accept new information from another person as trustworthy, generalizable, and relevant’ (Schroder‐Pfeifer et al., 2018, p. 330). Evidence suggests that goal‐oriented practices are acceptable to youth‐aged clients and their parents and carers (Jacob et al., 2022; O'Reilly et al., 2022; Stasiak et al., 2013). Evidence also shows that youth have reported that setting and reviewing goals helped them to stay on track in therapy, and that goals are easy or very easy for them to set (Pender et al., 2013). Links have been found between goal‐oriented practices with increased youth retention in therapeutic care (Cairns et al., 2019) and positive outcomes (Tryon et al., 2018). As such, there is clear interest and importance in providing clinical guidance around best practices that might promote positive long‐term outcomes for the growing number of youth seeking mental health support.

Since 2003, there has been an increased focus internationally on goal‐oriented practices in youth mental health settings (Cairns et al., 2019; Chiodo et al., 2022; O'Reilly et al., 2022). This is across the care continuum and in diverse support settings like schools (Chandrasekhar et al., 2023; Hartwig & Taylor, 2022; Spargo et al., 2021), community mental health clinics (Hurlburt et al., 2010; O'Reilly et al., 2022; Spinola et al., 2017) and intensive services (Balkin, Flores, & Casillas, 2011; Balkin, Leicht, et al., 2011; Hepper et al., 2005; Lee et al., 2018). While there are existing reviews focused on therapeutic goals with adults (Levack et al., 2015; Shick Tryon et al., 2018), an overview of idiographic goal‐based measures for all ages (Lloyd et al., 2019), and focused on specific elements of goals work, for example, therapeutic alliance (Jacob et al., 2022), as far as we are aware there is no broad systematic mapping of evidence focused on goal‐oriented practices for mental health support with youth.

This study aims to map the extent, range and nature of the evidence‐base for goal‐oriented practice for youth, including gaps pertaining to how goal‐oriented practice is defined and implemented in practice, and the features of mental health and wellbeing goal‐oriented practices in the contexts where evidence is available. The purpose is to guide future research on the development of effective strategies and tools to support goal‐oriented practices. The following questions guided the review: (1) What is the available empirical literature on mental health and wellbeing goal‐oriented practice with children and young people? (2) How are mental health and wellbeing goals defined and implemented with youth and parents/carers in practice (what is involved in the actual practice, e.g., was a tool used, was it goal setting or monitoring; and what strategies were used to implement goal oriented practices, e.g., was there training, any tools used to support the goals)? and (3) What are the features of mental health and wellbeing goal‐oriented practice with youth in the contexts where evidence is available?

## METHOD

Scoping reviews of a body of literature can be of particular use when the topic has not yet been extensively reviewed or is of a complex nature (Pham et al., 2014). For this reason, a scoping review of two decades of literature (2003–2023) was conducted following the Preferred Reporting Items for Systematic Reviews and Meta‐Analyses (PRISMA‐ScR) guidelines (Rethlefsen et al., 2021) and the Arksey and O'Malley framework (Arksey & O'Malley, 2005). See supplementary materials for the completed PRISMA checklist. The date range was deliberately selected to reflect current practices and emerging evidence to increase the applicability of results to contemporary healthcare settings (Helbach et al., 2022). The aim of a scoping review is to map the extent, range and nature of the literature on a topic of interest, as well as to determine possible gaps. Hence, it is a good methodological fit with the aims of this research.

Search terms were developed and agreed upon by all researchers and in collaboration with a health sciences librarian (LB) and peer‐reviewed following Peer Review of Electronic Search Strategies (PRESS) guidelines (McGowan et al., 2016). See Appendix A – Search Strategies. The project was registered on the open science framework (https://osf.io/kv46y/?view_only=cb5a301323cc4c54923198352b635ec9). A librarian conducted the search in MEDLINE (OVID), Embase (Elsevier), APA PsycINFO (EBSCOhost), Cochrane Database of Systematic Reviews (Wiley) and Scopus (Elsevier) on February 13, 2023. Results were limited from 2003‐current before export. No other limits or validated search filters were applied to the search. MEDLINE and Embase records were excluded in Scopus prior to export using the command *AND NOT ((INDEX(medline)) OR (INDEX(mbase)))*. All other duplicates were removed in EndNote using the method developed by Bramer et al. (2016).

The duplicated records were imported into Rayyan (Ouzzani et al., 2016) for title and abstract screening. Studies were included if they involved youth aged 0–18 years old (as an identified client, including parents and carers as relevant); described a goal‐oriented practice (including goal setting, goal‐based outcome monitoring), or goal setting (tasks and processes); focused on mental health and wellbeing (including substance use) related goal‐oriented practice; and provided specific descriptions of goal‐oriented practice outcomes, and/or experiences of clinicians and clients (including parents and carers). Both qualitative and quantitative studies were included. See Appendix B – Inclusion and Exclusion Criteria. Non‐empirical studies (e.g. opinion pieces and theoretical narratives) were excluded from the review to ensure the research built on the empirical evidence base, to move forward an evidence‐based understanding and interpretation of goal‐oriented practices where possible, and to identify key areas of evidence deficit. This aligns with the purpose of guiding future research on the development of evidence‐based strategies and tools to support goal‐oriented practices. Due to the volume of returned results, screening was completed in a two‐phase process involving seven reviewers (five senior researchers and two research assistants). First, 25% of all records were independently screened at the title and abstract level by a minimum of two reviewers. Disagreements were reviewed collaboratively and resolved through consensus and written instructions for coders were iteratively updated to tune inter‐rater agreement. Once 25% of studies were double‐coded, reviewed and the guidelines for screening refined, research assistants completed single‐screening of the remaining records. Consultation with broader research team members occurred where needed. Where a decision on inclusion was not possible at the abstract level (e.g. age range not reported) the article was moved to full‐text stage of screening. Researchers were not involved in the decisions to include or exclude studies that they had previously authored.

A codebook detailing the specific characteristics of interest to be extracted was developed and agreed upon by the authors. A standardized Excel form was used to extract data in the following six areas: (1) study identification (authors, title and key information), (2) methodology, (3) demographics, (4) clinical context, (5) dimensions of working with goals and (6) outcomes and experiences. Following methodological recommendations from Levac et al. (2010) the team of data extractors (two senior researchers and two research assistants) were trained as required and inducted into the research. Data extraction, collation, summarizing and reporting were iterative processes that used team dialogue and pilot testing (e.g. data extraction pilot tested on 10–15 studies with two reviewers) to promote consistent approaches to facilitate answering the research questions. We used a combination of descriptive numerical summaries and thematic analysis (Braun & Clarke, 2006, 2021) to explore the extent, nature and distribution of data. One researcher (LW) undertook a preliminary reading of the included studies and data extraction table and developed an initial thematic framework. Thematic analysis focused on reporting on patterns within the data either explicitly present or latent, and was derived not just from quantifiable coding but purposefully looking to develop new, co‐occurring or overarching themes and exploring their meanings. Multiple meetings and analysis reviews with other members of the research team (SC, DL, JJ and SH) and data extractors were held early in the thematic framework development to re‐assess and improve credibility and trustworthiness of the findings .

## RESULTS

**FIGURE 1:**
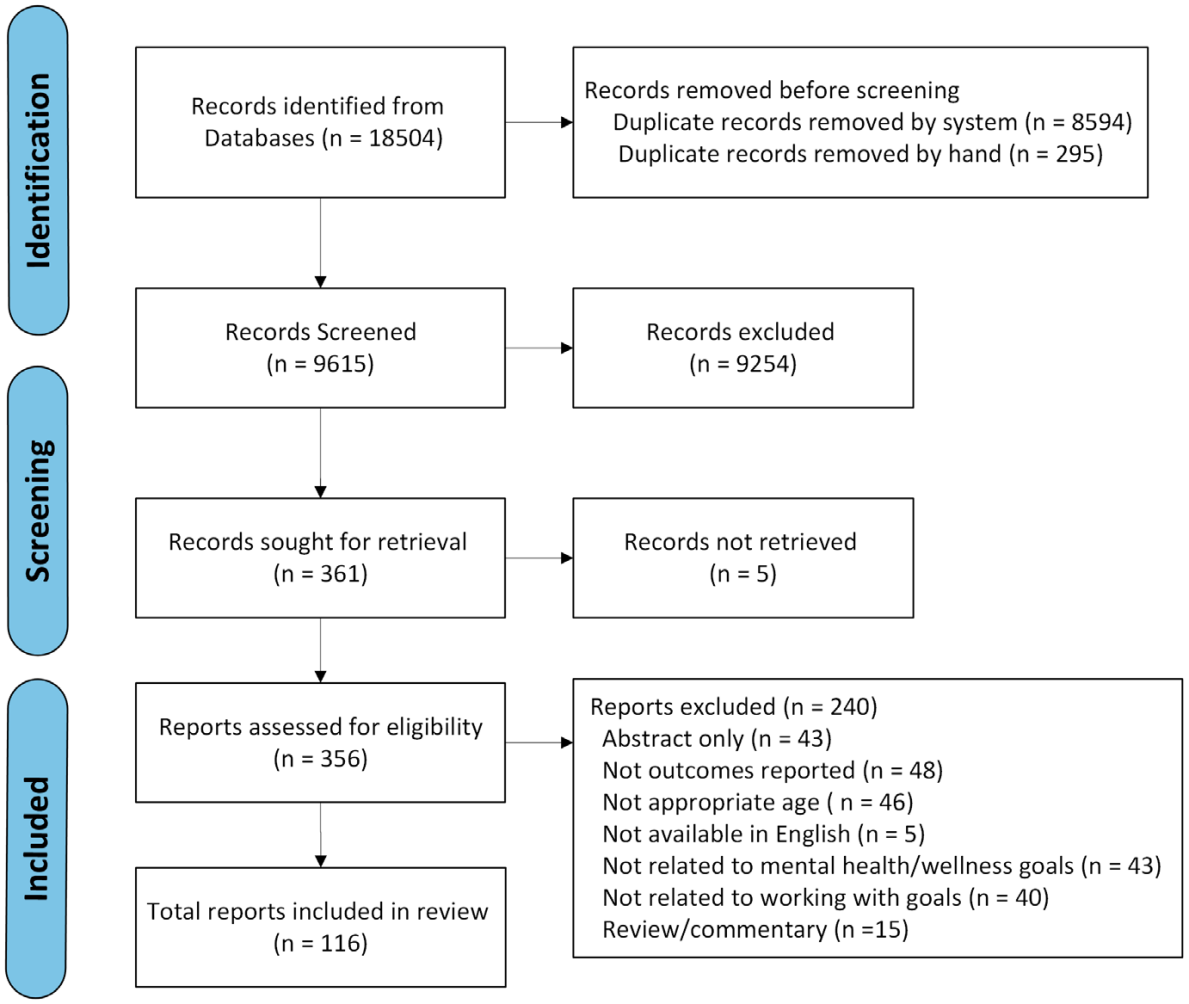
PRISMA flow diagram of report selection.

Searches of online databases of published materials generated a total of 18,504 records (see Figure 1 – PRISMA flow diagram).

After removing duplicates, a total of 9615 records were screened at the title and abstract level and 359 were assessed at full‐text stage. A total of 116 were included in the review. Just under half (47%, *n* = 55) were published since 2018, studies were conducted in 23 counties with the majority published in the United States (42%, *n* = 65) and United Kingdom (25%, *n* = 29). Most studies were quantitative (56%, *n* = 65) with primarily sample sizes of <100 (47%, *n* = 54). The studies were carried out in a variety of contexts including outpatient community mental health (24%, *n* = 28), schools (primary/elementary and secondary/middle/high publicly funded schools) (22%, *n* = 25), rehabilitation or inpatient treatment centres (13%, *n* = 15).

Study participants were primarily female (41%, *n* = 48) and between ages 12 and 18 (37%, *n* = 43). Participants were majority White in 34% (*n* = 39) of studies, in contrast to 16% (*n* = 19) of studies that had participants mainly from minoritized ethnic backgrounds (also referred to as Black and Indigenous People of Color: BIPOC). A diverse range in socioeconomic statuses were represented in 10% (*n* = 12) of studies, however, most studies did not report the socioeconomic status of participants or their families (75%, *n* = 87). Only 2% (*n* = 2) of studies included details regarding 2SLGBTQIA+ identities. Most studies reported that most youth participants lived with their family or caregiver (27%, *n* = 31), and several studies reported that participants had current or prior experience living in residential or foster care (19%, *n* = 22). Primary presenting problems varied across studies and included general mental health (17%, *n* = 31), autism (11%, *n* = 13), substance use (9%, *n* = 11), ADHD (3%, *n* = 4), depression (2%, *n* = 3), eating disorders (2%, *n* = 2) and multiple diagnoses (37%, *n* = 43). Please see Table 1.

**TABLE 1:** Characteristics of included articles.

Article	Sample size	Country	Design	Participant demographics								Service or intervention descriptors		
				Age range	Study population	Gender	Ethnicity	SES	2SLGBTQIA+	Presenting problems	Living arrangements	Setting	Therapeutic approach	Type of provider
McGoron et al. (2014)	101–500	United States	Empirical, journal article, quantitative (RCT)	<12, 12–18	Children, adolescents, parents	>50% female	>50% White	Not reported	Not reported	Primary (ADHD)	Not reported	Primary care	Not reported	Not reported
Alderson et al. (2019)	<100	United Kingdom	Empirical, journal article, qualitative	12–18, >18	Adolescents, young adults, caregivers, clinicians, social workers	>50% female	Not reported	Not reported	Not reported	Primary (substance use)	Family, foster care, residential care, independent, prior experience with care	Not reported	Motivational enhancement therapy, social behaviour and network therapy	Not reported
Alderson et al. (2020)	101–500	United Kingdom	Empirical, journal article, mixed methods (RCT)	12–18, >18	Adolescents, young adults, caregivers, clinicians, social workers	>50% female	>50% White	Low SES	Not reported	Primary (substance use)	Family, foster care, residential care, independent, prior experience with care	Community MHA	Motivational enhancement therapy, social behaviour and network therapy	Counsellor/therapist, psychologist, social worker
Arnold et al. (2007)	<100	United States	Empirical, journal article, qualitative	12–18	Adolescents	>50% female	>50% BIPOC	Low SES	Not reported	General mental wellness	Family, adoptive family/legal guardian	Other	Strengths‐based case management	Counsellor/therapist
Balkin, Flores, and Casillas (2011) and Balkin, Leicht, et al. (2011)	<100	United States	Empirical, journal article, quantitative	12–18	Adolescents	>50% female	>50% White	Not reported	Not reported	Primary (substance use)	Not reported	Residential treatment/hospital	Not reported	Counsellor/therapist, psychologist, social worker, nurse
Balkin, Flores, and Casillas (2011) and Balkin, Leicht, et al. (2011)	101–500	United States	Empirical, journal article, quantitative	12–18	Adolescents	>50% female	>50% White	Not reported	Not reported	Primary (depression)	Not reported	Residential treatment/hospital	Not reported	Counsellor/therapist, psychologist
Balkin and Roland (2007)	<100	United States	Empirical, journal article, quantitative	12–18	Adolescents	>50% female	Not reported	Not reported	Not reported	Primary (depression)	Not reported	Residential treatment/hospital	Not reported	Counsellor/therapist, psychologist, social worker
Balkin (2004)	<100	United States	Empirical, dissertation, quantitative	12–18	Adolescents	>50% female	Not reported	Not reported	Not reported	Multiple dx (depression, disruptive behaviour, substance use, psychosis, trauma, other)	Not reported	Residential treatment/hospital	Not reported	Counsellor/therapist, psychologist, social worker
Baudoin and Galand (2022)	501+	Belgium	Empirical, journal article, quantitative	12–18	Adolescents	>50% male	Not reported	Diverse SES	Not reported	General mental wellness	Family, residential care	K‐12 School	Not reported	Not reported
Becker et al. (2021)	<100	United States	Empirical, journal article, mixed methods	Not reported	Not reported	>50% female	>50% White	Not reported	Not reported	General mental wellness	Not reported	Community MHA	Not reported	Counsellor/therapist
Beery et al. (2017)	<100	United States	Empirical, journal article, quantitative	<12	Children	>50% male	>50% White	Middle SES	Not reported	Primary (ADHD)	Not reported	Other	Summer treatment program, behaviour modification interventions, medication	Not reported
Bhattacharya (2021)	<100	United States	Empirical, dissertation, mixed methods	12–18, >18	Adolescents, clinicians	>50% female	>50% White	Diverse SES	Not reported	Multiple dx (depression, anxiety, general mental wellness, other)	Not reported	Other	Behavioural activation, positive psychology, cognitive behavioural therapy	Counsellor/therapist, psychologist
Bögels et al. (2008)	<100	Netherlands	Empirical, journal article, quantitative	<12, 12–18	Children, adolescents, parents, caregivers	>50% male	Not reported	Not reported	Not reported	Multiple dx (ADHD, OCD, autism)	Family, adoptive family/legal guardian, prior experience with care	Community MHA	Mindfulness therapy, mindfulness training for parents	Psychologist
Bordelon and Bradley (2019)	N/A	United States	Empirical, book	Not reported	Not reported	Not reported	Not reported	Not reported	Not reported	Primary (autism)	Not reported	Not reported	Parent education and training in behaviour management, cognitive behaviour therapy, acceptance and commitment therapy, mindfulness‐based interventions	Not reported
Both Gragg (2006)	<100	United States	Empirical, dissertation, mixed methods (incl. case study)	12–18	Adolescents, parents, family members	Not reported	>50% BIPOC	Low‐middle SES	Not reported	Primary (substance use)	Family	Community MHA	Multidimensional family therapy, multisystemic family therapy, brief strategic family therapy	Counsellor/therapist, psychologist
Buckheit et al. (2018)	101–500	United States	Empirical, journal article, quantitative	12–18	Adolescents	>50% male	>50% White	Middle SES	Not reported	Primary (substance use)	Not reported	Community MHA	Cognitive behavioural therapy	Counsellor/therapist, psychologist
Cairns et al. (2019)	101–500	Australia	Empirical, journal article, quantitative	12–18, >18	Adolescents, young adults	>50% female	Not reported	Not reported	Not reported	Multiple dx (general mental wellness, substance use, other)	Not reported	Other	Not reported	Not reported
Cairns et al. (2015)	<100	Australia	Empirical, journal article, qualitative	12–18, >18	Adolescents, young adults	>50% female	Not reported	Not reported	Not reported	Multiple dx (general mental wellness, depression, anxiety, PTSD)	Family, independent	Community MHA	Not reported	Not reported
Carroll et al. (2013)	<100	Australia	Empirical, journal article, mixed methods (incl. case study)	12–18	Adolescents	>50% male	Not reported	Low SES	Not reported	Multiple dx (general mental wellness, substance use, other)	Family, foster care, prior experience with care	Other	Self‐regulatory intervention, cognitive behavioural therapy, strengths‐based strategies	Counsellor/therapist, social worker
Chandrasekhar et al. (2023)	101–500	United States	Empirical, journal article, quantitative	<12	Children	>50% female	>50% White	Not reported	Not reported	General mental wellness	Not reported	K‐12 School	Resilience‐building strategies	Health coach
Chang et al. (2023)	<100	Taiwan	Empirical, journal article, qualitative	Not reported	Not reported	Not reported	Not reported	Not reported	Not reported	Primary (eating disorder)	Not reported	Residential treatment/hospital	Not reported	Psychologist, physician, nurse, dietician
Choi et al. (2022)	101–500	United States	Empirical, journal article, quantitative	<12, 12–18	Children, adolescents, parents, caregivers	>50% male	>50% White	Not reported	Not reported	Primary (autism)	Not reported	Other	Applied behaviour analysis	Not reported
Churchman et al. (2021a, 2021b)	<100	United Kingdom	Empirical, journal article, mixed methods	<12, 12–18	Children, adolescents, parents, caregivers	>50% female	>50% White	Not reported	Not reported	General mental wellness	Not reported	K‐12 School	Method of levels therapy, parent–child activity (shared goals intervention)	Counsellor/therapist
Churchman et al. (2021a, 2021b)	<100	United Kingdom	Empirical, journal article, mixed methods (incl. case study)	<12, 12–18	Children, adolescents	>50% male	>50% White	Not reported	Not reported	General mental wellness	Not reported	K‐12 School	Method of levels therapy	Counsellor/therapist
Claessens et al. (2012)	501+	Uganda	Empirical, journal article, quantitative	<12	Children, parents	Not reported	Not reported	Not reported	Not reported	General mental wellness	Not reported	K‐12 School	Psychosocial life‐skills intervention	Social worker
Collins et al. (2023)	<100	Australia	Empirical, journal article, qualitative	<12, 12–18	Children, adolescents, parents	>50% female	Not reported	Not reported	Not reported	General mental wellness (co‐morbid to physical illness)	Family, foster care	Residential treatment/hospital	Not reported	Counsellor/therapist, psychologist
Davids et al. (2017)	101–500	South Africa	Empirical, journal article, quantitative	12–18	Adolescents	>50% female	>50% BIPOC	Diverse SES	Not reported	General mental wellness	Not reported	Not reported	Not reported	Not reported
Day et al. (2017)	N/A	United Kingdom	Empirical, journal article, mixed methods (RCT)	<12	Children, parents	Not reported	Not reported	Not reported	Not reported	Primary (parent with significant personality difficulties)	Family	Not reported	Parent education and training	Not reported
Dickson and Moberly (2010)	101–500	Australia	Empirical, journal article, quantitative	12–18, >18	Adolescents, young adults	>50% female	Not reported	Not reported	Not reported	Multiple dx (depression, anxiety, general mental wellness)	Not reported	K‐12 School	Not reported	Not reported
Eames et al. (2016)	<100	United Kingdom	Empirical, journal article, mixed methods	12–18	Adolescents	>50% male	>50% White	Not reported	Not reported	General mental wellness	Not reported	K‐12 School	Narrative therapy	Psychologist
Edbrooke‐Childs et al. (2015)	101–500	United Kingdom	Empirical, journal article, quantitative	<12	Children	>50% female	>50% White	Not reported	Not reported	Multiple dx (emotional disturbance, eating disorder, self‐harm, conduct disorder, learning difficulties, habit disorder, developmental difficulties, psychosis, general mental wellness, other)	Not reported	Other	Not reported	Not reported
Edmonson (2018)	<100	United States	Empirical, dissertation, quantitative	12–18	Adolescents	>50% female	>50% White	Not reported	Not reported	Primary (anxiety)	Not reported	Not reported	Not reported	Not reported
El‐Awad et al. (2017)	N/A	Germany	Empirical, journal article, narrative	12–18	Adolescents	Not reported	Not reported	Not reported	Not reported	General mental wellness	Unaccompanied refugees	Other	Not reported	Not reported
Everson (2018)	<100	United States	Empirical, dissertation, qualitative	Not reported	Not reported	>50% female	>50% White	Not reported	Not reported	Multiple dx (emotional disturbance, depression, anxiety, bipolar disorder, OCD, ADHD, other)	Not reported	K‐12 School	Individualized education plan (social skills instruction, parent and family involvement, behavioural management strategies, other)	Not reported
Fiks et al. (2013)	101–500	United States	Empirical, journal article, quantitative	<12	Children, parents, legal guardian	>50% male	>50% White	Upper SES	Not reported	Primary (ADHD)	Family, adopted family/legal guardian	Primary care	Behaviour therapy, medication	Not reported
Fiks et al. (2012)	101–500	United States	Empirical, journal article, quantitative	<12	Children, parents, clinicians	>50% male	>50% BIPOC	Upper SES	Not reported	Primary (ADHD)	Not reported	Not reported	Shared decision making, behaviour modification interventions, medication	Counsellor/therapist
Gabrielsen et al. (2012)	101–500	Norway	Empirical, journal article, quantitative	12–18, >18	Adolescents, young adults	>50% female	Not reported	Not reported	Not reported	General mental wellness	Not reported	Not reported	Not reported	Not reported
Gabrielsen et al. (2012)	101–500	Norway	Empirical, journal article, quantitative	12–18, >18	Adolescents, young adults	>50% female	Not reported	Not reported	Not reported	General mental wellness	Not reported	Not reported	Not reported	Not reported
Hartwig and Taylor (2022)	N/A	United States	Empirical, journal article, qualitative (incl. case study)	Not reported	Not reported	Not reported	Not reported	Not reported	Not reported	General mental wellness	Not reported	K‐12 School	Solution focused play therapy	Counsellor/therapist, social worker, teacher
Hepper et al. (2005)	<100	United Kingdom	Empirical, journal article, qualitative	<12	Children	>50% male	>50% White	Not reported	Not reported	Multiple dx (ADHD, OCD, autism, learning disability, depression, psychosis, anxiety)	Not reported	Residential treatment/hospital	Not reported	Not reported
Hinchey (2016)	<100	United States	Empirical, dissertation, quantitative	12–18	Adolescents	>50% male	>50% White	Low‐middle SES	Not reported	Multiple dx (emotional disturbance, depression, anxiety, ADHD, other)	Family, adopted family/legal guardian	K‐12 School	Solution focused brief therapy	Psychologist
Ho and Chen (2022)	101–500	China	Empirical, journal article, quantitative	12–18, >18	Adolescents, young adults	>50% female	>50% BIPOC	Diverse SES	Not reported	General mental wellness	Family	Other	Strength‐based parenting	Not reported
Hodgetts et al. (2018)	<100	Canada	Empirical, journal article, qualitative (incl. case study)	12–18	Adolescents, parents, clinicians, occupational therapists, nurses	>50% male	Not reported	Not reported	Not reported	Primary (autism)	Family	Not reported	Not reported	Not reported
Holbein et al. (2013)	101–500	United States	Empirical, journal article, quantitative	<12, 12–18	Children, adolescents, parents, caregivers	>50% female	>50% White	Diverse SES	Not reported	General mental wellness (co‐morbid with physical illness)	Not reported	Other	Camp‐based psychosocial intervention	Camp counsellor
Humphrey et al. (2010)	101–500	United Kingdom	Empirical, journal article, quantitative	<12	Children	>50% male	Not reported	Not reported	Not reported	General mental wellness	Not reported	K‐12 School	Cognitive behavioural therapy, social skills training, attribution training, parent education and training, teacher education and training	Teacher
Hurlburt et al. (2010)	<100	United States	Empirical, journal article, quantitative	<12	Children, parents, family members	>50% male	>50% White	Not reported	Not reported	Multiple dx (general mental wellness, ADHD, OCD, behavioural diagnosis, depression)	Not reported	Community MHA	Child and family therapy	Counsellor/therapist, psychologist, social worker
Imai et al. (2023)	<100	Japan	Empirical, journal article, mixed methods (incl. case study)	<12	Children	>50% male	Not reported	Not reported	Not reported	Primary (autism; developmental disorder, ADHD, other)	Not reported	Other	Not reported	Occupational therapist
Jacob et al. (2021)	501+	United Kingdom	Empirical, journal article, quantitative	<12, 12–18, >18	Children, adolescents, young adults, parents, caregivers, clinicians	>50% female	>50% White	Not reported	Not reported	General mental wellness	Not reported	Other	Not reported	Not reported
Jacob et al. (2022)	N/A	United Kingdom	Empirical, journal article, narrative	Not reported	Not reported	Not reported	Not reported	Not reported	Not reported	Multiple dx	Not reported	Other	Cognitive behavioural therapy	Counsellor/therapist, psychologist, physician, occupational therapist
Jacob et al. (2016)	101–500	United Kingdom	Empirical, journal article, qualitative	<12, 12–18	Children, adolescents, parents, clinicians	Not reported	Not reported	Not reported	Not reported	Multiple dx	Not reported	Other	Not reported	Not reported
Jacob, et al. (2017) and Jacob et al. (2015)	101–500	United Kingdom	Empirical, journal article, quantitative	<12, 12–18	Children, adolescents	Not reported	Not reported	Not reported	Not reported	Multiple dx	Not reported	Other	Not reported	Not reported
Kaminer et al. (2018)	101–500	United States	Empirical, journal article, quantitative	12–18	Adolescents	>50% male	>50% White	Not reported	Not reported	Primary (substance use)	Not reported	Not reported	Cognitive behavioural therapy	Counsellor/therapist, psychologist
Kanter et al. (2021)	101–500	United States	Empirical, journal article, quantitative	12–18	Adolescents	>50% female	>50% BIPOC	Diverse SES	Included	General mental wellness	Family, foster care, prior experience with care	Other	Youth relationship education program	Not reported
Kaplan and Steele (2005)	<100	United States	Empirical, journal article, quantitative	<12, 12–18, >18	Children, adolescents, young adults	>50% male	>50% White	Not reported	Not reported	Primary (autism)	Not reported	Community MHA	Music therapy	Counsellor/therapist, psychologist
Kiyimba et al. (2018)	<100	United Kingdom	Empirical, journal article, qualitative (incl. case study)	<12, 12–18	Children, adolescents, parents, family members, clinicians, other healthcare professionals	>50% male	Not reported	Not reported	Not reported	Multiple dx	Not reported	Community MHA	Not reported	Not reported
Kleinrahm et al. (2013)	101–500	Germany	Empirical, journal article, quantitative	<12, 12–18	Children, adolescents, caregivers	>50% male	Not reported	Low SES	Not reported	General mental wellness	Foster care, prior experience with care	Not reported	Not reported	Not reported
Kolehmainen et al. (2012)	501+	United Kingdom	Empirical, journal article, mixed methods	<12	Children, occupational therapists	Not reported	Not reported	Diverse SES	Not reported	General mental wellness	Not reported	Community MHA	Not reported	Occupational therapist
Krause et al. (2022)	501+	United Kingdom	Empirical, journal article, quantitative	12–18	Adolescents	>50% female	>50% White	Not reported	Not reported	Multiple dx (anxiety, depression)	Not reported	Community MHA	Not reported	Counsellor/therapist, psychologist
Lamash et al. (2023)	N/A	Israel	Empirical, journal article, qualitative	12–18, >18	Adolescents, young adults	Not reported	Not reported	Not reported	Not reported	Primary (autism)	Not reported	Not reported	Social participation and navigation intervention, positive psychology, self‐regulatory intervention	Psychologist, occupational therapist
Lavik et al. (2018)	<100	Norway	Empirical, journal article, qualitative	12–18, >18	Adolescents, young adults	>50% female	Not reported	Not reported	Not reported	Multiple dx (anxiety, depression, trauma, self‐harm, suicidal ideation, other)	Not reported	Community MHA	Systemic and family therapy, cognitive behavioural therapy, mentalisation‐based therapy	Counsellor/therapist, psychologist, psychiatrist
Law (2022)	N/A	United Kingdom	Empirical, journal article, narrative	Not reported	Not reported	Not reported	Not reported	Not reported	Not reported	Multiple dx (trauma, general mental wellness)	Not reported	Not reported	Not reported	Counsellor/therapist
Lee et al. (2018)	101–500	United Kingdom	Empirical, journal article, quantitative	12–18	Children, clinicians, caregivers	>50% female	Not reported	Not reported	Not reported	Multiple dx	Not reported	Residential treatment/hospital	Family therapy, individual therapy (different models), medication	Counsellor/therapist, psychologist, psychiatrist, social worker, nurse, occupational therapist
Luebbe et al. (2018)	101–500	China	Empirical, journal article, quantitative	12–18	Adolescents, parents, family members	>50% female	>50% BIPOC	Diverse SES	Not reported	Multiple dx (anxiety, general mental wellness)	Not reported	K‐12 School	Not reported	Not reported
Mallion and Wood (2020)	N/A	United Kingdom	Empirical, journal article, narrative	12–18	Adolescents	Not reported	Not reported	Not reported	Not reported	Multiple dx (substance use, depression, anxiety, suicidality, other)	Not reported	Other	Functional family therapy, cognitive behavioural therapy, multisystemic therapy	Counsellor/therapist
Marttinen and Salmela‐Aro (2012))	501+	Finland	Empirical, journal article, quantitative	12–18, >18	Adolescents, young adults	>50% male	Not reported	Not reported	Not reported	General mental wellness	Not reported	K‐12 School	Not reported	Not reported
Matalí et al. (2020)	101–500	Spain	Empirical, journal article, quantitative	12–18	Adolescents	>50% male	Not reported	Not reported	Not reported	Primary (substance use)	Not reported	Community MHA	Not reported	Not reported
Maybery et al. (2013)	<100	United Kingdom	Empirical, journal article, quantitative	<12, 12–18	Children, adolescents, parents	Not reported	Not reported	Not reported	Not reported	Multiple dx (families with substance use and/or mental health problems)	Family, adopted family/legal guardian	Community MHA	Not reported	Counsellor/therapist, psychologist, social worker
Mazzotti et al. (2012)	<100	United States	Empirical, journal article, quantitative	<12	Children	>50% male	>50% BIPOC	Not reported	Not reported	Multiple dx (ADHD, learning disability, behaviour difficulties, developmental disabilities, other)	Not reported	K‐12 School	Not reported	Teacher
McCarthy and McDevitt (2018)	<100	Ireland	Empirical, journal article, qualitative	<12, 12–18	Children, adolescents, parents	>50% female	Not reported	Diverse SES	Not reported	Multiple dx (conduct disorder, anxiety, depression, eating disorder, other)	Not reported	Community MHA	Not reported	Not reported
McCarthy et al. (2010)	<100	United Kingdom	Empirical, journal article, quantitative	12–18	Adolescents	>50% female	Not reported	Not reported	Not reported	General mental wellness	Not reported	Other	Psychological skills training	Psychologist
Müller et al. (2023)	<100	United States	Empirical, journal article, mixed methods	<12	Children	>50% male	>50% White	Not reported	Not reported	Primary (autism)	Not reported	K‐12 School	Not reported	Teacher
Mounts and Kim (2007)	<100	United States	Empirical, book	12–18	Adolescents, parents, caregivers	>50% female	>50% White	Low‐middle SES	Not reported	General mental wellness	Not reported	Other	Not reported	Not reported
O'Dell et al. (2020)	101–500	United States	Empirical, journal article, quantitative	12–18	Adolescents	>50% female	>50% White	Not reported	Not reported	Multiple dx (ADHD, depression, adjustment disorder, anxiety, conduct disorder, other)	Not reported	Primary care	Acceptance and commitment therapy, medication	Psychologist
Odhammar and Carlberg (2015)	<100	Sweden	Empirical, journal article, qualitative	<12	Children	>50% male	Not reported	Not reported	Not reported	Multiple dx (ADHD, disruptive behaviour disorders, anxiety, general mental wellness)	Not reported	Other	Psychodynamic therapy	Counsellor/therapist, psychologist, social worker
O'Reilly et al. (2022)	501+	Ireland	Empirical, journal article, quantitative	12–18, >18	Adolescents, young adults	>50% female	Not reported	Not reported	Not reported	Multiple dx	Family, independent, foster care, residential home, other	Community MHA	Not reported	Not reported
Paré and Marcotte (2021)	<100	Canada	Empirical, journal article, quantitative	>18	Young adults	>50% female	>50% White	Not reported	Not reported	Multiple dx (depression, anxiety, general mental wellness)	Family, independent, other	University	Not reported	Not reported
Penno et al. (2022)	<100	New Zealand	Empirical, journal article, qualitative	12–18, >18	Adolescents, young adults	>50% female	Not reported	Not reported	Included	Multiple dx (depression, anxiety, other)	Not reported	Other	Cognitive behavioural therapy, interpersonal therapy, mentalisation‐based therapy, dialectical behaviour therapy, acceptance and commitment therapy.	Counsellor/therapist, psychologist, psychiatrist
Pratt (2022)	<100	United States	Empirical, dissertation, mixed methods	12–18	Adolescents	>50% male	>50% BIPOC	Not reported	Not reported	General mental wellness	Court‐involved; previously spent time in detention, on probation, residential facility	Other	Not reported	Not reported
Pretorius et al. (2018)	<100	United Kingdom	Empirical, journal article, mixed methods (incl. case study)	<12	Children, caregivers	>50% male	>50% BIPOC	Low SES	Not reported	Multiple dx	Family, foster care, prior experience with care	Community MHA	Caregiver‐child psychotherapy, individual child psychotherapy	Counsellor/therapist, psychologist, other mental health staff
Qureshi (2018)	<100	United States	Empirical, dissertation, qualitative (incl. case study)	12–18	Adolescents	>50% male	>50% BIPOC	Low SES	Not reported	Multiple dx (conduct disorder, ADHD, PTSD/trauma, mood disorders, anxiety, OCD, eating disorders, other)	Family, prior experience with care	Residential treatment/hospital	Family therapy; multidimensional family therapy, brief strategic family therapy, attachment‐based family therapy	Counsellor/therapist
Randell et al. (2022)	101–500	United Kingdom	Empirical, journal article, mixed methods (RCT)	<12	Children, caregivers, therapists, occupational therapists	>50% male	>50% White	Not reported	Not reported	Primary (autism; sensory processing difficulties)	Not reported	Other	Sensory integration therapy	Counsellor/therapist
Ranzato et al. (2021)	<100	United Kingdom	Empirical, journal article, qualitative	<12, 12–18	Children, adolescents, foster carers	>50% female	Not reported	Not reported	Not reported	General mental wellness	Foster care, prior experience with care	Other	Not reported	Not reported
Rodger and Vishram (2010)	<100	Australia	Empirical, journal article, qualitative (incl. case study)	<12	Children	>50% male	Not reported	Not reported	Not reported	Primary (autism)	Not reported	Community MHA	Cognitive orientation to daily occupational performance	Counsellor/therapist
Rouquette et al. (2021)	101–500	France	Empirical, journal article, quantitative	<12, 12–18	Children, adolescents	>50% male	Not reported	Not reported	Not reported	General mental wellness	Not reported	Other	Not reported	Not reported
Rupani et al. (2014)	<100	United Kingdom	Empirical, journal article, mixed methods	12–18	Adolescents	>50% female	>50% White	Not reported	Not reported	Multiple dx	Not reported	K‐12 School	Not reported	Counsellor/therapist
Salter et al. (2016)	<100	Australia	Empirical, journal article, quantitative	<12	Children	>50% male	>50% White	Not reported	Not reported	Primary (autism)	Family	Community MHA	Child‐centred play therapy	Counsellor/therapist
Sanders et al. (2023)	N/A	United States	Empirical, journal article, narrative	<12	Children	Not reported	Not reported	Not reported	Not reported	General mental wellness	Not reported	K‐12 School	Social and emotional learning skills interventions	Teacher
Schmit (2016)	<100	United States	Empirical, dissertation, quantitative	12–18	Adolescents	>50% female	>50% BIPOC	Not reported	Not reported	Multiple dx (depression, bipolar disorder, psychosis, PTSD, adjustment disorder, substance use, other)	Not reported	Residential treatment/hospital	Not reported	Counsellor/therapist
Smith (2015)	<100	United States	Empirical, dissertation, quantitative	12–18	Adolescents	>50% male	>50% BIPOC	Not reported	Not reported	Multiple dx (suicidality, behavioural difficulties, psychosis, other)	Not reported	Residential treatment/hospital	Existential questioning, rational emotive behavioural therapy, cognitive behavioural therapy, dialectical behavioural therapy, motivational interviewing, solution focused therapy, narrative therapy, family therapy	Counsellor/therapist, psychologist
Spargo et al. (2021)	<100	United States	Empirical, journal article, quantitative	12–18, >18	Adolescents, young adults	>50% male	>50% BIPOC	Not reported	Not reported	Primary (substance use)	Family, legal guardian	K‐12 School	Psychoeducational program, social/emotional program	Counsellor/therapist, teacher
Spinola et al. (2017)	101–500	United States	Empirical, journal article, quantitative	12–18	Adolescents	>50% male	>50% White	Not reported	Not reported	Primary (substance use)	Family, legal guardian	Community MHA	Not reported	Not reported
Suarez‐Balcazar et al. (2022)	<100	United States	Empirical, journal article, qualitative (incl. case study)	12–18	Adolescents	>50% male	>50% BIPOC	Low SES	Not reported	Primary (autism)	Family	Community MHA	Not reported	Not reported
Suldo and Doll (2020)	N/A	United States	Empirical, book	12–18	Adolescents	Not reported	Not reported	Not reported	Not reported	General mental wellness (plus psychopathology)	Not reported	K‐12 School	Not reported	Counsellor/therapist
Taurogiński et al. (2022)	101–500	Poland	Empirical, journal article, qualitative	12–18	Adolescents, parents, family members	>50% female	Not reported	Not reported	Not reported	Multiple dx (eating disorder, conduct disorder, depression, adjustment disorder, anxiety, psychosis, other)	Family, legal guardian	Community MHA	Family therapy	Counsellor/therapist, psychologist, psychiatrist
Veltro et al. (2015)	101–500	Italy	Empirical, journal article, quantitative	12–18	Adolescents	>50% female	Not reported	Not reported	Not reported	General mental wellness	Not reported	K‐12 School	Not reported	Psychologist
Vieselmeyer (2018)	101–500	United States	Empirical, dissertation, quantitative	12–18	Adolescents	>50% female	>50% White	Diverse SES	Not reported	General mental wellness	Family	Not reported	Sport‐based youth development program	Not reported
Wahl et al. (2011)	N/A	Germany	Empirical, journal article, narrative	<12, 12–18	Children, adolescents	Not reported	Not reported	Not reported	Not reported	Primary (depression)	Not reported	K‐12 School	Not reported	Not reported
Walworth (2007)	101–500	United States	Empirical, journal article, quantitative	<12, 12–18, >18	Children, adolescents, young adults	>50% male	Not reported	Not reported	Not reported	Primary (autism)	Not reported	Community MHA	Music therapy	Counsellor/therapist
Walworth et al. (2009)	<100	United States	Empirical, journal article, quantitative	<12, 12–18, >18	Children, adolescents, young adults	Not reported	Not reported	Not reported	Not reported	Primary (autism)	Not reported	Community MHA	Music therapy	Counsellor/therapist
Werch et al. (2010)	101–500	United States	Empirical, journal article, quantitative	12–18	Adolescents	>50% female	>50% BIPOC	Diverse SES	Not reported	General mental wellness	Not reported	K‐12 School	Not reported	Nurse, health education specialist
Wolpert et al. (2015)	N/A	United Kingdom	Empirical, journal article, narrative	Not reported	Not reported	Not reported	Not reported	Not reported	Not reported	Multiple dx	Not reported	Residential treatment/hospital	Not reported	Counsellor/therapist, psychologist, other mental health staff
Wolpert et al. (2012)	501+	United Kingdom	Empirical, journal article, quantitative	<12, 12–18	Children, adolescents	>50% male	Not reported	Not reported	Not reported	Multiple dx (emotional disorder, conduct disorder, autism, ADHD, other)	Not reported	Community MHA	Not reported	Not reported
Wong et al. (2021)	<100	Singapore	Empirical, journal article, quantitative	<12, 12–18	Children, adolescents	>50% male	>50% BIPOC	Not reported	Not reported	General mental wellness (co‐morbid with physical illness)	Not reported	Residential treatment/hospital	Music therapy, psychosocial and supportive care	Counsellor/therapist, psychologist, occupational therapist
Wynne et al. (2016)	<100	Ireland	Empirical, journal article, quantitative	12–18	Adolescents	>50% female	Not reported	Not reported	Not reported	Multiple dx	Family	Community MHA	Family therapy intervention	Psychologist, psychiatrist, social worker, nurse, occupational therapist, speech and language therapist
Zaitsoff et al. (2015)	<100	Canada	Empirical, journal article, qualitative	12–18	Adolescents	>50% female	>50% White	Middle‐upper SES	Not reported	Primary (eating disorder)	Not reported	Other	Motivational interviewing	Counsellor/therapist
Cooper and Law (2018)	N/A	United Kingdom	Empirical, book	Not reported	Not reported	Not reported	Not reported	Not reported	Not reported	General mental wellness	Not reported	Not reported	Not reported	Counsellor/therapist, psychologist, other mental health staff
Cox et al. (2010)	101–500	United States	Empirical, journal article, quantitative	<12, 12–18	Children, adolescents	>50% male	>50% White	Diverse SES	Not reported	Multiple dx (general mental wellness, anxiety, depression, ADHD, psychosis, substance use, other)	Family, prior experience with care, residential care, previously court‐involved	Community MHA	Not reported	Not reported
Cronin and Allen (2015)	101–500	United Kingdom	Empirical, journal article, quantitative	<12, 12–18	Children, adolescents	>50% male	Not reported	Not reported	Not reported	General mental wellness	Not reported	Sports organization	Not reported	Coach
Derrick (2018)	101–500	United States	Empirical, dissertation, mixed methods	Not reported	Not reported	Not reported	Not reported	Low‐middle SES	Not reported	Multiple dx	Family, legal guardian, caregiver, prior experience with care	Residential treatment/hospital	Not reported	Not reported
DiBartolo and Varner (2012)	101–500	United States	Empirical, journal article, quantitative	<12	Children	>50% female	>50% White	Not reported	Not reported	Multiple dx (anxiety, depression, general mental wellness)	Not reported	Not reported	Not reported	Not reported
Dickens (2020)	<100	United States	Empirical, book	12–18	Adolescents, parents, family members	>50% male	>50% BIPOC	Not reported	Not reported	Multiple dx (depression, eating disorder)	Family	Community MHA	Family therapy, narrative therapy	Counsellor/therapist
Dickson and MacLeod (2006)	<100	Australia	Empirical, journal article, quantitative	12–18	Adolescents	>50% male	Not reported	Not reported	Not reported	General mental wellness	Not reported	K‐12 School	Not reported	Not reported
Fellows (2003)	101–500	United States	Empirical, dissertation, quantitative	12–18	Adolescents	>50% female	>50% BIPOC	Middle SES	Not reported	General mental wellness	Not reported	K‐12 School	Not reported	Not reported
Godley et al. (2008)	<100	United States	Empirical, journal article, quantitative	12–18	Adolescents	>50% male	>50% White	Not reported	Not reported	Primary (substance use)	Not reported	Residential treatment/hospital	Contingency management, multisystemic therapy intervention	Not reported
Grant (2021)	101–500	United States	Empirical, dissertation, quantitative	>18	Young adults	>50% male	>50% White	Not reported	Not reported	Primary (substance use)	Not reported	University	Not reported	Not reported
Hollmann et al. (2018)	501+	Germany	Empirical, journal article, quantitative	<12, 12–18	Children, adolescents, parents	>50% male	Not reported	Not reported	Not reported	General mental wellness	Family, legal guardian, caregiver	K‐12 School	Not reported	Not reported

*Note*: Gender coded as binary variable (> or <50% female participants), referring to children/adolescents/young adults only; Ethnicity coded as binary variable (> or>50% BIPOC participants), referring to children/adolescents/young adults only; SES (i.e. socioeconomic status) refers to that of majority of participants or families or diverse representation of socioeconomic status within study population; 2SLGBTQIA+ refers to any mention of gender‐diversity within study population, Type of Provider refers to counsellor/therapist as Master's level training, psychologist refers to PhD level clinician.

### How are mental health and wellbeing goals defined and implemented with youth and parents or Carers in practice?

Multiple therapeutic approaches were reported by 20% (*n* = 23) of the studies. The therapeutic approach was not reported in just over half of the studies (51%, *n* = 59). Where therapeutic approach was reported, the most commonly reported therapeutic approach was various family therapy models (15%, *n* = 17), followed by cognitive behavioural therapy (9%, *n* = 10). Multiple types of providers were listed in 27% of studies (*n* = 31) and Master's level counsellors or therapists were the most common service provider (40%, *n* = 46). Type of provider was not reported by 43% (*n* = 50) of studies. 74 studies (64%) did not provide information on practitioner or researcher training or stated that training was not required. Of the remaining studies (*n* = 42, 36%), there were six levels of goal training: (a) Training was stated as pertaining to some element of goal setting, striving or goal‐oriented practice by 3% (*n* = 4) studies; (b) Workshops about elements of goal‐oriented practice were used as training in 3% (*n* = 3) of studies; (c) Specific goal‐based measure training was stated in 7% (*n* = 8) of studies; (d) Training in specific measures that are not goal focused was stated in 8% (*n* = 9) of studies; (e) Training not specific to goals (e.g. drug and alcohol work, CBT, solution focused therapy) was stated in 10% (*n* = 12) of studies and (f) Unspecified training was reported in 5% (*n* = 6) of studies.

### Individuals involved in goal setting

Most studies (66%, *n* = 76) described goal setting as being primarily led by youth. In addition, goals were described as being primarily led by parents or caregivers in 10% (*n* = 12) of studies and in 7% (*n* = 8) of studies goals were jointly agreed. In 6% (*n* = 7) of studies, goals were described as led by counsellors or clinicians. In the remaining 3% (*n* = 4) of studies, families, other instructors, or an app were described as primarily involved in setting the goals. There were nine (8%) studies that did not indicate who was primarily involved in setting the goals.

### Components of goal‐oriented practices

Just over half of the studies were focused only on goal setting (51%, *n* = 59), and many focused only on goal attainment (16%, *n* = 19). Seven (6%) studies focused on both goal setting and goal attainment. The timeline for goal setting was described as taking place during a single session in 30% (*n* = 35) of studies. The follow up timepoint in these studies was either not defined or there was no further follow up time point to check progress towards goals. Goal setting and tracking were described as taking place over a period of time (i.e. more than a single session) in 62% (*n* = 72) of studies. This ranged from between 1 week and 12 months follow up time points. The remaining nine studies (8%) did not define the follow up.

### Tools used to facilitate goal‐oriented practices

Five (4%) studies described technology or apps used to record goals and/or monitor the progress of goals. These goals included bespoke social goals (tracked through bespoke goal measures), top problem goals, idiographic treatment goals and social or organizational goals (goal measure not described). Four (3%) studies focused on goal importance, striving and engagement. Two (2%) studies focused solely on goal‐based measurement. In terms of goal‐based measures, 72 (62%) studies described the use of goal‐based measures. Multiple goal‐based measures were described by four (4%) studies. Table 2 provides a breakdown of the reported measures.

**TABLE 2:** Breakdown of measures described and corresponding number of studies.

Measures used	Number of studies
Achievement Goal Questionnaire – Revised	1
Adolescent Life Goal Profile Scale (ALGPS)	2
Adolescent Substance Abuse Goal Commitment (ASAGC) Questionnaire	2
Aspirations Index	1
Athletic Coping Skills Inventory (ACSI‐28; goal setting subscale)	1
Bespoke goal measures or questions	15
Canadian Occupational Performance Measure (COPM)	2
Collaboration on Treatment Goals and Tasks Scale	1
Goal and Plans Task	2
Goal Attainment Scale (GAS)	12
Goal Attainment Scale of Stabilization (GASS)	5
Goal Based Outcomes Tool (GBO)	17
Goals Task Measures	1
Personal Achievement Goal Orientation scales of the Patterns of Adaptive Learning Scales (PALS)	1
Personal Best Scale	1
Personal Project Analysis (PPA)	4
Pre‐Task Predictions Questionnaire	1
SCERTS model	1
Self‐Determined Learning Model of Instruction (SDLMI)	1
State Hope Scale	1
The Goal Attainment Measure (GAM)	1
Thoughts About Abstinence Scale	1
Top Problems	2
Total	76

Of the 72 studies that reported the use of goal‐based measures, 14 (19%) reported positive changes in goal ratings at a follow‐up time point. Goals that were progressed towards achievement were reported to be focused on mental health symptoms, confidence, social skills, social connectedness, management of self‐care, independence, emotional skills, mental health knowledge, personal goals, accommodation needs and academic goals. Only a small number of studies reported any deterioration in goal progress (Edbrooke‐Childs et al., 2015; Jacob et al., 2021).

Reflections from a small number of experiences of goal‐oriented practices were present in the included studies. For example, the importance of ensuring a broad view of goal setting as used in practice was highlighted in a study that emphasized setting goals in addition to substance use goals in their support (Alderson et al., 2019). Simultaneously considering goal types, for example, personal goals and classroom goal structures using multilevel models (Baudoin & Galand, 2022) was also suggested as an aligned way to enhance goal‐oriented practice.

One study reported that clients preferred an online format to track goals (Bhattacharya, 2021). No other studies discussed experiences of using goal‐based outcome measures, nor made recommendations about their use, or advised for which populations they are most suited.

### What are the features of mental health and wellbeing goal‐oriented practice in these contexts?

Close reading of included studies identified three main themes and a total of six subthemes (See Table 3). These themes, described below, are interpretive constructs intended to go beyond the primary studies and generate new ways of framing features of the goal‐oriented practice literature.

**TABLE 3:** Key themes.

Themes	Subthemes
Theme 1: Conceptual and empirical constructs of goal‐oriented practices	(a) Typologies, taxonomies and hierarchies (b) Labels and language (c) Multiple sites and sources of goal‐talk (d) Representation of goals and progress
Theme 2: Dimensions and intersections of ‘quality’ in goal‐oriented practice	(a) Goal agreement and fidelity (b) Interventions to improve the quality of goals
Theme 3: The socio‐cultural contexts of goal‐oriented practice	

#### Theme 1: Conceptual and empirical constructs of goal‐oriented practices

This theme explores the tensions of terminology and construct definition around goal‐oriented practices. It also covers the complexities of conceptualizing mental wellness‐related goal‐practices within the broader goal‐talk happening in the lives of youth through four sub‐themes.

##### Typologies, taxonomies and hierarchies

There were a variety of ways in which primary authors proposed or applied goal typologies (i.e. conceptual heuristics), goal taxonomies (i.e. empirical measures) and goal hierarchies (i.e. ordering of categories into levels) in their study designs or analysis. Examples of approaches to goal typologies included both dichotomous distinctions between categories like intrinsic and extrinsic goals (Hollmann et al., 2018), mastery versus performance goals (Baudoin & Galand, 2022) or emotional/mental well‐being versus personal growth/social development goals (Everson, 2018); as well as polytomous distinctions like social communication, emotional regulation, or transactional goals (Walworth et al., 2009). Multiple studies explicitly utilized the SMART goal criteria (Specific, Measurable, Achievable, Relevant, Time‐Bound) (e.g. Bhattacharya, 2021; Cairns et al., 2015, 2019) as a guideline for quality. In other instances, goals were conceptualized as connected to discrete areas of a youth's life. For example, in an RCT exploring reinforcement of personal goal activities for adolescents with substance use disorders, youth were prompted to select goals in one of 10 areas (e.g. social/recreational, educational; Godley et al., 2008).

##### Labels and language

Overall, the language and terminology used to differentiate or define goal‐oriented practices were rarely linked explicitly to theory or construct definitions. This lack of coherent understanding was reflected in the wide range of labels to describe what youth and families were working towards: ‘life goals’, ‘wellness goals’, ‘personal goals’, ‘self‐improvement goals’, ‘treatment goals’ and ‘future goals’. While some researchers (Gabrielsen et al., 2013) positioned goal‐setting within ‘recovery‐oriented’ language (i.e. the aim of goal‐setting is the pursuit of purpose and meaning, and believing that life indeed can become meaningful, where goals protect the individual against hopelessness and despair); there was considerable emphasis across studies with performance‐oriented language around working with goals which centred on ‘goal‐striving’, ‘goal‐attainment’, ‘goal achievement’ and ‘goal‐directedness’ as ways of describing key behaviours and mechanisms of change.

##### Multiple sites and sources of goal‐talk

Importantly, while the majority of studies (63%, *n* = 73) included a focus on setting goals related to symptoms, psychosocial functioning and behaviours, many goal‐oriented practices related to mental health and substance use were embedded in or complimentary to broader goal‐talk and strategies targeting personal academic achievement (Fiks et al., 2013), physical rehabilitation (Imai et al., 2023), career planning (Ranzato et al., 2021), sports performance (McCarthy et al., 2010), and other life skills and experiences. Studies often explored correlations and connections within and across mental wellness goals, or other types of goals and various behavioural outcomes. For example, in a study by Paré and Marcotte (2021), students in the intervention group with a more positive appraisal of their academic and career goals were reported to show fewer depressive symptoms than students in the intervention group with a less positive appraisal of these goals.

##### Representation of goals and goal Progress

One feature of goal‐oriented practices across the context of the studies was the diverse ways of representing goals and movement or progress towards (or away from) goals. The most typical approach focused on quantifying goals numerically through both formulating a set number of goals (e.g. setting three goals at the beginning of treatment, Eames et al., 2016), and using rubrics or linear sliding scales to score and quantify the rate the amount of progress made in achieving goals over time (Salter et al., 2016). Encouraging youth to write out their goals in their own words as opposed to just verbalizing them was noted as a mechanism to promote engagement, commitment, and increase focus (Cooper & Law, 2018; Matalí et al., 2020; McCarthy et al., 2010). Less often, authors described how to incorporate visual representations as part of goal‐oriented practices. Werch et al. (2010) employed an image‐based strategy for messages sent to youth to increase health behaviour goal setting and reduce substance use that was particularly useful among older adolescents who use substances. In a more recent study, by Hartwig and Taylor (2022) involving solution focused play therapy, the sandtray approach was used, where children engaged in goal setting. This was primarily by constructing a physical picture of their goals through representation of people, objects, thoughts and behaviours rather than words (Hartwig & Taylor, 2022).

#### Theme 2: Quality and making ‘good’ goals

This theme highlights the complexity surrounding how to define high‐quality goal‐oriented practices through two sub‐themes, including interventions that were developed and tested to improve goal quality prior to engaging in therapeutic support and how goal‐based data might be a change driver within health services generally.

##### Goal agreement and Fidelity

Given the complex therapeutic relationships that underpin goal‐oriented practices, it was not surprising that across the body of literature, a consistent feature of working with goals was the complex role of the clinician in continually negotiating and attuning understandings about the goals of interest between different people. Sometimes this mediation facilitated negotiation (the process of the client negotiating their goals, supported by the clinician) was focused on dyads of youth‐therapist (Hartwig & Taylor, 2022) and youth‐parent (Mayberry‐et al., 2013). In other instances, goal‐planning involved a team of multiple healthcare providers (e.g. Penno et al., 2022 reports a psychiatrist, psychologist and outreach worker in addition to youth and family involved). In these instances, it was the clinician who facilitated soliciting input and reflection from numerous people contributing to the overall success of the youth's treatment, ultimately resulting in better effects of treatment (e.g. Bordelon & Bradley, 2019). In addition to complex processes related to agreement on goals, there was also evidence of low agreement between clinicians' self‐ratings of goal‐oriented practice and their observed fidelity (i.e. degree to which they implemented as intended, assessed by researchers). In one study, congruence between therapists' self‐rating of goal‐oriented activities during sessions with youth and third‐party objective observers was explored (Hurlburt et al., 2010). Therapists reported pursuing 2.5 times more goals and strategies per session, on average, than identified by third‐party trained coders. Correspondence between therapists and coders about the occurrence of specific goals and strategies in treatment sessions was low, with only 20.5% of codes having a Kappa of .4 or higher.

##### Interventions to improve the quality of goals

While some studies explored the impact of goal‐oriented practices on mental health outcomes (i.e. effectiveness), others measured the impact of interventions designed to improve the quality of the goals youth set (i.e. optimization). Several studies proposed protocolized interventions to improve goal‐quality in advance of initiating the therapeutic intervention – suggesting a kind of pre‐intervention for goal‐setting was deemed necessary for positive mental health outcomes. For example, the ‘IvySCIP Goal Builder’ was evaluated in the study by Müller et al. (2023). This is an online tool that supports student lesson plans, reports, and personalized goals. A comparison of goals before and after the use of the IvySCIP goal builder found that targeted behaviours were clearly specified in 83.5% of goals before the introduction of the goal builder, but clearly specified in 100% of goals after; and measurement criteria were clearly specified in 46.8% of goals prior to the introduction of the goal builder, but clearly specified in 97% of goals after introduction. In a study by Kolehmainen et al. (2012), a learning module to support teens with generating ideas for SMART goals was developed and evaluated. The youth could then plan their SMART goals by being directed to another learning module. There was also some evidence that goal‐based outcome measures were used, not only to assess individual client progress and inform individual treatment planning, but as data to support health service planning. In a recent and novel study by Jacob et al. (2023), findings suggested that idiographic measures may have some utility alongside more standardized measures in informing service improvement and may be of help in that they provide different perspectives and areas of important focus. The study explored how movement towards goals might serve as a proxy indicator of service change overall.

#### Theme 3: The socio‐cultural contexts of goal‐oriented practice

This theme explores the intersections of individual participant and community context features that characterize the literature reviewed. Overall, reporting on the key sociodemographic factors of participants was sparse, limiting what we know and can say about goal‐oriented practices for specific sub‐populations within specific sociocultural contexts. Specifically, there was a pattern of not reporting on key determinants of mental health outcomes including 2SLGBTQIA+, socioeconomic status (SES), ethnicity and sex or gender identity. Where demographic data were reported, they suggested that most studies to date have involved participants who are White, identify as female, and from mixed SES populations. A small number of studies involved primarily study participants from minoritized ethnic (BIPOC) backgrounds. Goal‐oriented practices related to mental health appeared to be delivered in a range of settings, with a range of providers in a range of community spaces (i.e. schools, community mental health clinics and community centres). Country of study origin provides only limited insight into cultural characteristics. Despite these complex intersectional environments, a minority of studies reported any kind of intervention adaptations for working with goals in equity‐deserving populations specifically (e.g. translation of assessments or instructions into another language, culturally safe practices or approaches, age adaptations).

## DISCUSSION

This review aimed to scope the existing empirical literature for goal‐oriented practices with youth, including identifying gaps in how goal‐oriented practice is defined and implemented, and the features of mental health and wellbeing goal‐oriented practices in these contexts. This review provides the first synthesis of the available empirical evidence from the past 20 years. Through the process of reviewing the 116 included studies and exploring common themes and gaps, this scoping review has identified considerable variation in how goal‐oriented practices are defined, operationalized, and measured in mental health and wellbeing contexts pertaining to youth.

Collectively, the literature included in this review has several strengths and shortcomings. The literature provides a detailed and nuanced picture of the relatively complex concept of goal‐oriented practices, which is a considerable strength. This includes the use of goal typologies, taxonomies, and hierarchies to organize and order goals that are set in the course of therapy. Alongside the use of SMART goal criteria (Bhattacharya, 2021) used in three studies, numerous ways of grouping, nesting or differentiating goals was described. Despite the majority of studies being focused only on goal setting, the language used to describe goal‐oriented practices and goal setting was also diverse. The breadth of interpretation of goal‐oriented practices and how they are therefore implemented is demonstrated in the lack of relations to theory or prior evidence in the empirical literature. While there was also variation in who leads goal setting, the majority of studies described goals as being led by youth, with the clinician acting as a facilitator to goal formulation, which aligns with the shared decision‐making ethos of goal‐oriented practices (Law, 2022).

While there were inconsistencies in the level of detail provided across the studies, there is substantial scope for future research to build on the shortcomings of this literature and issues that have been overlooked. Most pertinently, the majority of the available evidence relating to goal‐oriented practices with youth are from Western countries, primarily engaging with adolescent, female, White participants, and who were not from diverse socioeconomic backgrounds (where data were available). This homogeneity risks maintaining the focus of research and mental health knowledge from these groups and lessens the knowledge and voices from participants majority world contexts, who are from genders aside from female and from minoritized ethnic (BIPOC) backgrounds. Future research should focus on goal‐oriented practices with groups that are less represented here, including those whose mental health is supported by voluntary, community and social enterprise organizations.

The vast majority of studies did not specify what goal‐oriented practice training was provided or stated that training was not necessary. This might be because goal‐focused work was included in broader training provided (e.g. CBT), but this is unclear from the available evidence. For the studies that indicated that practitioners received training in specific goal‐based measures used as a tool for supporting goal formulation as well as tracking outcomes, it would further knowledge in this area to explore how these measures were adhered to in practice. As a wider point of reflection, no studies discussed the use of a goal‐oriented practice fidelity tool. Only one study (Dickson & Moberly, 2010) mentioned any exploration of fidelity to goal formulation, where researchers cross‐checked participants' goals to ensure they understood the corresponding goal setting matrix provided. There was also a lack of recommendations specific to the use of goal‐oriented practices with youth from the studies themselves; that is, no indications of best practice were identified from the included studies. It would be beneficial for future research to start to explore goal‐oriented practices in more depth to gain a deeper understanding of how goal‐oriented practices are implemented and corresponding outcomes. The development of (a) a set of practice principles and (b) a fidelity to model tool would also be highly beneficial in this regard. The broad description and understanding of goal‐oriented practices, as evident in this literature review, could be streamlined through a set of practice principles, to aid clinician training, and to ensure consistent interpretation and practice. A fidelity to model tool could then be used to assess how far practice aligns, or not, with a given set of practice principles. This would contribute to consistent and reliable practice moving forward. From there, more robust research could explore what elements of goal‐oriented practices are effective in the pursuit of positive mental health and wellbeing outcomes, under what circumstances, and for whom.

An aligned area for further exploration relates to the inconsistencies across studies in the use of goal‐based tools to assist the formulation and progress tracking of goals. The majority of studies reported the use of bespoke goal measures, with 12 further measures only being used in one study each. The most commonly used tool was the Goal‐Based Outcomes tool (GBO, Law, 2013), which has psychometric properties reported (Duncan et al., 2023; Edbrooke‐Childs et al., 2015). Only a small proportion of studies that reported the use of goal‐based measures also reported outcome information. The vast majority of such reported outcomes that were in a positive direction towards goal achievement, and in a diverse range of outcome domains. This speaks to the personalized nature and flexibility of goal‐oriented practice, and the importance of exploring key areas of outcome as defined by youth (Jacob et al., 2017, 2015). However, future research should focus on exploring any associations between goal outcomes, types of goals, and the elements of goal setting with different populations in various settings. As the evidence evolves, a systematic review or meta‐analysis will, in time, be possible and could answer more questions about correlations and causality than a scoping review is currently able to. This future research would move the field further along in its understanding of what aspects of goal‐oriented practice are effective and less effective for youth. Previous research has touched on this topic (e.g. Jacob et al., 2022) but was not able to link to types of goals or areas of positive and negative outcome.

Where tracking goals was employed, a range of ways of recording this was described, including primarily numeric tracking, but also rubrics or sliding scales and visualizations of progress. There is a recent move towards consideration of outcome measurement for a range of participants (see CORC, 2023). This includes an increase in accessibility considerations, which is particularly pertinent when centring youth in their own care and outcomes, as goal‐oriented practices do. In other research, efforts have also been made to synthesize the reporting of goal types, such that overarching goal ‘core concepts’ have been derived from existing research that reported goals formulated by and with youth using the GBO tool (Mok et al., 2024). In this study, the researchers conducted a review of existing taxonomies that are used to categorize goals, analysing them to create an overarching four ‘core concepts’ that encapsulate the previously developed taxonomy categories. The use of ‘core concepts’ may be of value for the consideration and analysis of future goals. Future research should consider how goal tracking is presented and grouped for analysis and reflection purposes to further support the streamlining of the work across settings and with diverse participant groups. Further, a minority of studies described the use of technology to record and track goals. All used bespoke goal‐based tools. Future research should seek to explore the benefits and drawbacks of using different goal tools as well as the use of technology to support goal‐oriented practices. Alignment in the use of tools may be conducive to future research to better understand goal‐oriented practices and goal outcomes.

There are strengths and limitations to this review. Strengths include the thorough database searching, broad inclusion criteria, and the diverse views and perspectives of multiple researchers. However, engaging a large group of screeners and reviewers may have impacted the consistency of decision‐making. Further limitations include the omission of grey literature and non‐empirical branching, and the researchers being outside the age range of the research focus, limiting interpretations and grounding in age‐related lived experience. As scoping reviews focus primarily on synthesizing the breadth of evidence, we recognize the limited depth in our analysis or fewer direct implications for decision‐makers. As this field of research matures, systematic reviews and meta‐analysis will be important to explore effect size across multiple studies and theorizing more deeply about sources of heterogeneity. Future primary studies should conduct stratified analyses and identify sociodemographic characteristics of youth, as is known to impact mental health therapy engagement and outcomes. Consistently defined outcomes and populations will facilitate helpful future meta‐analysis.

## CONCLUSION

This scoping review aims to synthesize the existing empirical evidence to guide future research on strategies and tools to support goal‐oriented practices. The findings demonstrate inconsistencies in reporting and measuring goal‐oriented practices, which in turn leaves a gap in empirical evidence for supporting youth mental health difficulties through goal‐oriented practices. Several areas for future research have been highlighted that will build on this evidence and further understanding in this area. Crucially, work towards the development of best practice principles will move practice towards uniformity in its understanding and delivery of goal‐oriented practices.

## AUTHOR CONTRIBUTIONS

**Jenna Jacob:** Conceptualization; data curation; formal analysis; investigation; methodology; project administration; supervision; validation; writing – original draft; writing – review and editing. **Lori Wozney:** Conceptualization; investigation; funding acquisition; writing – original draft; methodology; validation; visualization; writing – review and editing; formal analysis; project administration; supervision; data curation. **Hanne Weie Oddli:** Conceptualization; funding acquisition; investigation; methodology; validation; writing – review and editing. **Charlie Duncan:** Conceptualization; investigation; methodology; validation; writing – review and editing; supervision. **Jill Chorney:** Conceptualization; investigation; validation; writing – review and editing; supervision; methodology. **Debbie Emberly:** Conceptualization; investigation; methodology; validation; supervision; writing – review and editing. **Duncan Law:** Conceptualization; investigation; methodology; validation; writing – review and editing; supervision. **Sharon Clark:** Conceptualization; investigation; methodology; validation; writing – review and editing; supervision. **Sofie Heien:** Conceptualization; investigation; methodology; validation; writing – review and editing; supervision. **Leah Boulos:** Methodology; data curation; validation; writing – original draft; writing – review and editing. **Mick Cooper:** Conceptualization; investigation; writing – review and editing; methodology; validation; supervision.

## FUNDING INFORMATION

This work was partially funded by Canadian Institutes of Health Research Grant No. 186495.

## CONFLICT OF INTEREST STATEMENT

Jenna Jacob works on the Child Outcomes Research Consortium (CORC) project, based at Anna Freud, which encourages the use of outcome measures in youth mental health and wellbeing settings. Charlie Duncan is employed by the British Association for Counselling and Psychotherapy which is a membership body for counselling professionals in the UK. No other authors report any conflict of interest.

## Supporting information


Data S1.

## Data Availability

INSERT STATEMENT HERE (Templates available at: https://authorservices.wiley.com/author‐resources/Journal‐Authors/open‐access/data‐sharing‐citation/data‐sharing‐policy.html).

## APPENDIX A SEARCH STRATEGIES

**Table:**

MEDLINE all (Ovid)		Embase (Elsevier Embase.com)		PsycINFO (EBSCOhost)		CDSR (Wiley)		Scopus (Elsevier Scopus.com)	
1	exp Mental Disorders/ [NB: includes exp. Substance‐Related Disorders/]	1	‘mental disease’/exp	1	DE ‘Mental Disorders’ OR DE ‘Affective Disorders’ OR DE ‘Anxiety Disorders’ OR DE ‘Bipolar Disorder’ OR DE ‘Borderline States’ OR DE ‘Chronic Mental Illness’ OR DE ‘Dissociative Disorders’ OR DE ‘Eating Disorders’ OR DE ‘Gender Dysphoria’ OR DE ‘Mental Disorders due to General Medical Conditions’ OR DE ‘Neurocognitive Disorders’ OR DE ‘Neurodevelopmental Disorders’ OR DE ‘Neurosis’ OR DE ‘Obsessive Compulsive Disorder’ OR DE ‘Paraphilias’ OR DE ‘Personality Disorders’ OR DE ‘Psychosis’ OR DE ‘Serious Mental Illness’ OR DE ‘Sleep Wake Disorders’ OR DE ‘Somatoform Disorders’ OR DE ‘Stress and Trauma Related Disorders’ OR DE ‘Substance Related and Addictive Disorders’ OR DE ‘Thought Disorders’	1	MeSH descriptor: [Mental Disorders] explode all trees	1	TITLE‐ABS‐KEY(adhd OR ‘attention deficit’ OR anorexi* OR bulimi* OR ((bing* OR eating OR feeding OR purg*) W/2 disorder*) OR anxiety OR depress* OR ((behavio* OR mental* OR mood OR psychiatr* OR psycholog* OR stress) W/2 (condition* OR difficult* OR distress* OR health* OR disorder* OR ill OR illness* OR patient* OR problem* OR symptoms OR trauma*)) OR ((developmental OR sexual) W/2 trauma*) OR ((mental OR psychiatric OR psychological) W/3 (wellbeing OR ‘well‐being’)) OR ‘post‐traumatic stress’ OR ‘posttraumatic stress’ OR ptsd OR psychos* OR psychotic OR schizophreni* OR ‘self harm*’ OR ‘self injur*’ OR suicid* OR addict* OR ((alcohol OR drug* OR narcotic* OR substance*) W/2 (abus* OR depend* OR disorder* OR habit* OR misus* OR ‘use’ OR user OR users OR using)) OR alcoholic* OR alcoholism OR ‘binge drink*’ OR intoxicat* OR (medication* W/3 (abus* OR misus*)))
2	Mental Health/	2	‘mental health’/exp	2	DE ‘Mental Health’	2	MeSH descriptor: [Mental Health] this term only		
3	exp Mental Health Services/	3	‘mental health service’/exp	3	DE ‘Mental Health Services’ OR DE ‘College Mental Health Services’ OR DE ‘Community Mental Health Services’ OR DE ‘Psychological First Aid’ OR DE ‘School Based Mental Health Services’	3	MeSH descriptor: [Mental Health Services] explode all trees		
4	(adhd or attention deficit).ti,ab,kf.	4	(adhd OR ‘attention deficit’):ti,ab,kw	4	TI(adhd OR ‘attention deficit’) OR AB(adhd OR ‘attention deficit’)	4	(adhd or attention deficit)		
5	(anorexi* or bulimi* or ((bing* or eating or feeding or purg*) adj2 disorder*)).ti,ab,kf.	5	(anorexi* OR bulimi* OR ((bing* OR eating OR feeding OR purg*) NEAR/2 disorder*)):ti,ab,kw	5	TI(anorexi* OR bulimi* OR ((bing* OR eating OR feeding OR purg*) N2 disorder*)) OR AB(anorexi* OR bulimi* OR ((bing* OR eating OR feeding OR purg*) N2 disorder*))	5	(anorexi* or bulimi* or ((bing* or eating or feeding or purg*) near/2 disorder*))		
6	(anxiety or depress*).ti,ab,kf.	6	(anxiety OR depress*):ti,ab,kw	6	TI(anxiety OR depress*) OR AB(anxiety OR depress*)	6	(anxiety or depress*)		
7	((behavio?r* or mental* or mood or psychiatr* or psycholog* or stress) adj2 (condition* or difficult* or distress* or health* or disorder* or ill or illness* or patient* or problem* or symptoms or trauma*)).ti,ab,kf.	7	((behavio$r* OR mental* OR mood OR psychiatr* OR psycholog* OR stress) NEAR/2 (condition* OR difficult* OR distress* OR health* OR disorder* OR ill OR illness* OR patient* OR problem* OR symptoms OR trauma*)):ti,ab,kw	7	TI((behavio#r* OR mental* OR mood OR psychiatr* OR psycholog* OR stress) N2 (condition* OR difficult* OR distress* OR health* OR disorder* OR ill OR illness* OR patient* OR problem* OR symptoms OR trauma*)) OR ((behavio#r* OR mental* OR mood OR psychiatr* OR psycholog* OR stress) N2 (condition* OR difficult* OR distress* OR health* OR disorder* OR ill OR illness* OR patient* OR problem* OR symptoms OR trauma*))	7	((behavio?r* or mental* or mood or psychiatr* or psycholog* or stress) near/2 (condition* or difficult* or distress* or health* or disorder* or ill or illness* or patient* or problem* or symptoms or trauma*))	

	8	((developmental or sexual) adj2 trauma*).ti,ab,kf.	8	((developmental OR sexual) NEAR/2 trauma*):ti,ab,kw	8	TI((developmental OR sexual) N2 trauma*) OR AB((developmental OR sexual) N2 trauma*)	8		((developmental or sexual) near/2 trauma*)
	9	((mental or psychiatric or psychological) adj3 (wellbeing or well‐being)).ti,ab,kf.	9	((mental OR psychiatric OR psychological) NEAR/3 (wellbeing OR ‘well‐being’)):ti,ab,kw	9	TI((mental OR psychiatric OR psychological) N3 (wellbeing OR ‘well‐being’)) OR AB((mental OR psychiatric OR psychological) N3 (wellbeing OR ‘well‐being’))	9		((mental or psychiatric or psychological) near/3 (wellbeing or well‐being))
	10	(post‐traumatic stress or posttraumatic stress or ptsd).ti,ab,kf.	10	(‘post‐traumatic stress’ OR ‘posttraumatic stress’ OR ptsd):ti,ab,kw	10	TI(‘post‐traumatic stress’ OR ‘posttraumatic stress’ OR ptsd) OR AB(‘post‐traumatic stress’ OR ‘posttraumatic stress’ OR ptsd)	10		(post‐traumatic stress or posttraumatic stress or ptsd)
	11	(psychos?s or psychotic or schizophreni*).ti,ab,kf.	11	(psychos?s OR psychotic OR schizophreni*):ti,ab,kw	11	TI(psychos?s OR psychotic OR schizophreni*) OR AB(psychos?s OR psychotic OR schizophreni*)	11		(psychos?s or psychotic or schizophreni*)
	12	(self harm* or self injur*).ti,ab,kf.	12	(‘self harm*’ OR ‘self injur*’):ti,ab,kw	12	TI(‘self harm*’ OR ‘self injur*’) OR AB(‘self harm*’ OR ‘self injur*’)	12		(self harm* or self injur*)
	13	suicid*.ti,ab,kf.	13	suicid*:ti,ab,kw	13	TI(suicid*) OR AB(suicid*)	13	suicid*
14	or/1–13 [mental health]	14	#1 OR #2 OR #3 OR #4 OR #5 OR #6 OR #7 OR #8 OR #9 OR #10 OR #11 OR #12 OR #13	14	S1 OR S2 OR S3 OR S4 OR S5 OR S6 OR S7 OR S8 OR S9 OR S10 OR S11 OR S12 OR S13	14	#1 or #2 or #3 or #4 or #5 or #6 or #7 or #8 or #9 or #10 or #11 or #12 or #13	

	15	addict*.ti,ab,kf.	15	addict*:ti,ab,kw	15	TI(addict*) OR AB(addict*)	15		addict*
	16	((alcohol or drug* or narcotic* or substance*) adj2 (abus* or depend* or disorder* or habit* or misus* or ‘use’ or user? or using)).ti,ab,kf.	16	((alcohol OR drug* OR narcotic* OR substance*) NEAR/2 (abus* OR depend* OR disorder* OR habit* OR misus* OR ‘use’ OR user$ OR using)):ti,ab,kw	16	TI((alcohol OR drug* OR narcotic* OR substance*) N2 (abus* OR depend* OR disorder* OR habit* OR misus* OR ‘use’ OR user# OR using)) OR AB((alcohol OR drug* OR narcotic* OR substance*) N2 (abus* OR depend* OR disorder* OR habit* OR misus* OR ‘use’ OR user# OR using))	16		((alcohol or drug* or narcotic* or substance*) near/2 (abus* or depend* or disorder* or habit* or misus* or ‘use’ or user? or using))
	17	(alcoholic* or alcoholism or binge drink*).ti,ab,kf.	17	(alcoholic* OR alcoholism OR ‘binge drink*’):ti,ab,kw	17	TI(alcoholic* OR alcoholism OR ‘binge drink*’) OR AB(alcoholic* OR alcoholism OR ‘binge drink*’)	17		(alcoholic* or alcoholism or binge drink*)
	18	intoxicat*.ti,ab,kf.	18	intoxicat*:ti,ab,kw	18	TI(intoxicat*) OR AB(intoxicat*)	18		intoxicat*
	19	(medication* adj3 (abus* or misus*)).ti,ab,kf.	19	(medication* NEAR/3 (abus* OR misus*)):ti,ab,kw	19	TI(medication* N3 (abus* OR misus*)) OR AB(medication* N3 (abus* OR misus*))	19		(medication* near/3 (abus* or misus*))
	20	or/15–19 [substance use]	20	#15 OR #16 OR #17 OR #18 OR #19	20	S15 OR S16 OR S17 OR S18 OR S19	20		#15 or #16 or #17 or #18 or #19
	21	14 or 20	21	#14 OR #20	21	S14 OR S20	21	#14 or #20
22	Goals/		‐	22	DE ‘Goals’ OR DE ‘Goal Setting’	22	MeSH descriptor: [Goals] this term only		
23	((addiction? or behavio?r* or mental or psychiatric or psychological or substance*) adj3 (intervention* or practice* or rehab* or service* or therap* or treatment*)).ti,ab,kf.		‐	23	TI((addiction# OR behavio#r* OR mental OR psychiatric OR psychological OR substance*) N3 (intervention* OR practice* OR rehab* OR service* OR therap* OR treatment*)) OR AB((addiction# OR behavio#r* OR mental OR psychiatric OR psychological OR substance*) N3 (intervention* OR practice* OR rehab* OR service* OR therap* OR treatment*))	23	((addiction? or behavio?r* or mental or psychiatric or psychological or substance*) near/3 (intervention* or practice* or rehab* or service* or therap* or treatment*))		
24	(counselling or counselling).ti,ab,kf.		‐	24	TI(counselling OR counselling) OR AB(counselling OR counselling)	24	(counselling or counselling)		
25	or/23–24			25	S23 OR S24	25	#23 or #24		
26	22 and 25			26	S22 AND S25	26	#22 and #25		
27	(goal? adj5 (counselling or counselling or intervention* or practice* or rehab* or service* or therap* or treatment*)).ti,ab,kf.	22	(goal$ NEAR/5 (counselling OR counselling OR intervention* OR practice* OR rehab* OR service* OR therap* OR treatment*)):ti,ab,kw	27	TI(goal# N5 (counselling OR counselling OR intervention* OR practice* OR rehab* OR service* OR therap* OR treatment*)) OR AB(goal# N5 (counselling OR counselling OR intervention* OR practice* OR rehab* OR service* OR therap* OR treatment*))	27	(goal? near/5 (counselling or counselling or intervention* or practice* or rehab* or service* or therap* or treatment*))	2	TITLE‐ABS‐KEY((goal* W/5 (counselling OR counselling OR intervention* OR practice* OR rehab* OR service* OR therap* OR treatment*)) OR (goal* W/1 (set OR setting OR personal*)) OR ((aim OR goal* OR task) W/1 (based OR directed OR oriented)))
28	(goal? adj1 (set or setting or personal*)).ti,ab,kf.	23	(goal$ NEAR/1 (set OR setting OR personal*)):ti,ab,kw	28	TI(goal# N1 (set OR setting OR personal*)) OR AB(goal# N1 (set OR setting OR personal*))	28	(goal? near/1 (set or setting or personal*))		
29	((aim? or goal? or task?) adj (based or directed or oriented)).ti,ab,kf.	24	((aim$ OR goal$ OR task$) NEAR/1 (based OR directed OR oriented)):ti,ab,kw	29	TI((aim# OR goal# OR task#) N1 (based OR directed OR oriented)) OR AB((aim# OR goal# OR task#) N1 (based OR directed OR oriented))	29	((aim? or goal? or task?) near/1 (based or directed or oriented))		
30	or/27–29	25	#22 OR #23 OR #24	30	S27 OR S28 OR S29	30	#27 or #28 or #29		
31	21 and 30	26	#21 AND #25	31	S21 AND S30	31	#21 and #30		
32	26 or 31		‐	32	S26 OR S31	32	#26 or #31		
33	exp Child/ or Child Health Services/ or Paediatrics/ or (child* or kid or kids or girl or girls or boy or boys or adolescent* or adolescence or teen* or young person* or young people or young adult* or youth* or youngster* or preschool* or pre‐school* or kindergarten* or school* or student* or pupil* or juvenile* or minors or p?ediatric*).ti,ab,kf.	27	‘child’/exp. OR ‘child health care’/de OR ‘paediatrics’/de OR ‘child psychiatry’/de OR (child* OR kid OR kids OR girl OR girls OR boy OR boys OR adolescent* OR adolescence OR teen* OR ‘young person*’ OR ‘young people’ OR ‘young adult*’ OR youth* OR youngster* OR preschool* OR ‘pre‐school*’ OR kindergarten* OR school* OR student* OR pupil* OR juvenile* OR minors OR p$ediatric*):ti,ab,kw	33	DE ‘Paediatrics’ OR TI(child* OR kid OR kids OR girl OR girls OR boy OR boys OR adolescent* OR adolescence OR teen* OR ‘young person*’ OR ‘young people’ OR ‘young adult*’ OR youth* OR youngster* OR preschool* OR ‘pre‐school*’ OR kindergarten* OR school* OR student* OR pupil* OR juvenile* OR minors OR p#ediatric*) OR AB(child* OR kid OR kids OR girl OR girls OR boy OR boys OR adolescent* OR adolescence OR teen* OR ‘young person*’ OR ‘young people’ OR ‘young adult*’ OR youth* OR youngster* OR preschool* OR ‘pre‐school*’ OR kindergarten* OR school* OR student* OR pupil* OR juvenile* OR minors OR p#ediatric*)	33	MeSH descriptor: [Child] explode all trees	3	TITLE‐ABS‐KEY(child* OR kid OR kids OR girl OR girls OR boy OR boys OR adolescent* OR adolescence OR teen* OR ‘young person*’ OR ‘young people’ OR ‘young adult*’ OR youth* OR youngster* OR preschool* OR ‘pre‐school*’ OR kindergarten* OR school* OR student* OR pupil* OR juvenile* OR minors OR paediatric* OR paediatric*)
34	32 and 33	28	#26 AND #27	34	S32 AND S33	34	MeSH descriptor: [Child Health Services] this term only		combine using search history
35	limit 34 to yr. = ’2003 ‐Current’	29	#26 AND #27 AND [2003–2023]/py		limit results 2003‐current	35	MeSH descriptor: [Paediatrics] this term only		remove MEDLINE and EMBASE using AND NOT ((INDEX(medline)) OR (INDEX(embase)))
						36	(child* or kid or kids or girl or girls or boy or boys or adolescent* or adolescence or teen* or young person* or young people or young adult* or youth* or youngster* or preschool* or pre‐school* or kindergarten* or school* or student* or pupil* or juvenile* or minors or p?ediatric*)		
	**2513 results 2023‐02‐13**		**4442 results 2023‐02‐13**		**4793 results 2023‐02‐13**	37	#33 or #34 or #35 or #36		**6029 results 2023‐02‐13**
						38	#32 and #37		4553 records removed in Scopus
							limit Jan 1, 2003 to current		**1476 records exported 2023‐02‐13**
							**727 results 2023‐02‐13**		

## APPENDIX B INCLUSION AND EXCLUSION CRITERIA

**Table:**

Inclusion criteria	Exclusion criteria
Date: Published from 2003 onwards. Language: Available in English. Participants: Children and young people aged 0–18 years old (as identified client, including caregivers as relevant). Intervention/Practice: Goal‐oriented practice (including goal setting, goal‐based outcome monitoring), or goal setting (tasks and processes). Condition: Mental health and wellbeing (including substance use). Outcome: Provided specific descriptions of goal‐oriented practice outcomes, experiences of clinicians and clients (including caregivers) of goal‐oriented practices, goal outcomes. Sources Design: Qualitative, quantitative studies, systematic case studies, descriptive studies of services.	Sources focused on working with goals in health‐related contexts that are not specifically related to mental health, for example, the use of goals in physical injury rehabilitation. Sources that only reference primary studies but do not report on original data (i.e. frequently book chapters, therapeutic manuals and policy reports). Abstracts where only limited information about the study could be captured.
